# A Hybrid Geometric Spatial Image Representation for scene classification

**DOI:** 10.1371/journal.pone.0203339

**Published:** 2018-09-12

**Authors:** Nouman Ali, Bushra Zafar, Faisal Riaz, Saadat Hanif Dar, Naeem Iqbal Ratyal, Khalid Bashir Bajwa, Muhammad Kashif Iqbal, Muhammad Sajid

**Affiliations:** 1 Department of Software Engineering, Mirpur University of Science & Technology, Mirpur, Azad-Kashmir, Pakistan; 2 Department of Computer Science, Government College University, Faisalabad, Pakistan; 3 Department of Computer Science & IT, Mirpur University of Science & Technology, Mirpur, Azad-Kashmir, Pakistan; 4 Department of Electrical Engineering, Mirpur University of Science & Technology, Mirpur, Azad-Kashmir, Pakistan; 5 Faculty of Computer and Information Systems, Islamic University of Madinah, Madinah, Kingdom of Saudi Arabia; 6 Department of Mathematics, Government College University, Faisalabad, Pakistan; University of Science and Technology Beijing, CHINA

## Abstract

The recent development in the technology has increased the complexity of image contents and demand for image classification becomes more imperative. Digital images play a vital role in many applied domains such as remote sensing, scene analysis, medical care, textile industry and crime investigation. Feature extraction and image representation is considered as an important step in scene analysis as it affects the image classification performance. Automatic classification of images is an open research problem for image analysis and pattern recognition applications. The Bag-of-Features (BoF) model is commonly used to solve image classification, object recognition and other computer vision-based problems. In BoF model, the final feature vector representation of an image contains no information about the co-occurrence of features in the 2D image space. This is considered as a limitation, as the spatial arrangement among visual words in image space contains the information that is beneficial for image representation and learning of classification model. To deal with this, researchers have proposed different image representations. Among these, the division of image-space into different geometric sub-regions for the extraction of histogram for BoF model is considered as a notable contribution for the extraction of spatial clues. Keeping this in view, we aim to explore a Hybrid Geometric Spatial Image Representation (HGSIR) that is based on the combination of histograms computed over the rectangular, triangular and circular regions of the image. Five standard image datasets are used to evaluate the performance of the proposed research. The quantitative analysis demonstrates that the proposed research outperforms the state-of-art research in terms of classification accuracy.

## 1 Introduction

The category-wise classification of digital images is considered as one of the main requirement in computer vision applications such as scene analysis, remote sensing, medical science and image retrieval [1–7]. The changes in scale, illumination, rotations, overlapping objects, appearance of same view in the images of different classes, complex structures and difference in image spatial atterns make image classification an open research problem [8]. In past, global spatial features such as color and texture were used to perform image classification [1]. The low computational cost and simple implementation were considered as the main advantages of global spatial features [1]. In recent years, the Bag-of-Features (BoF) model is applied in various domains to perform image classification and scene analysis [1]. In BoF model, the local features [9] are extracted, quantized in the feature space and a histogram-based representation is used for image representation [9]. Feature extraction, feature description, codebook generation and order-less representation of image in the form of histograms of visual word are considered as the main steps of BoF model [8]. The lack of spatial information in histogram-based image representation is considered a limitation of BoF model [10–12].

The approaches based on a larger codebook size, query expansion and soft quantization are applied to enhance the classification accuracy of BoF model [11, 13]. The main limitation of all these approaches is the lack of spatial information that is considered to be beneficial for image classification-based problems [10, 11]. Researchers have proposed different forms of image representations to address this problem [10–12, 14–16]. In a broader way, the approaches that are applied for the computation of sematic spatial layout for histogram-based image representation are divided into two groups [11]: i) computation of spatial information through geometric relationships/ co-occurrences of visual words [14, 16, 17] ii) division of image into geometric sub-regions such as rectangles [10], triangles [11, 13] and circles [12]. The approaches based on geometric sub-division of image for histogram computation are reported robust as compared to the approaches based on geometric relationships among visual words [14]. In the case of geometric relationships [14, 16], the computational complexity increases with the size of code-book due to increase in the number of geometric relations among visual words [16].

In the first group [14, 16–18], the spatial information is computed by using the co-occurrences of visual words or by exploring the geometric relationships among them in the 2-D image space [14]. In these approaches [16], the geometric relationships among the words are computed by using a reduced size of codebook, as the relationships among words decreases due to increase in the size of codebook. Khan et al. [14] computed the global spatial information by computing the histograms of Pairs of Identical Words (PIWs), that are based on the angles among the same cluster/visual word. The histogram-based spatial representation of Khan et al. [14] is reported robust to the changes in scale and translation. In another research [17], Triplets of Identical Visual Words (TIWs) are computed to achieve rotation invariant image representation by calculating angles among three visual words. Savarese et al. [18] explored the spatial information among visual words by representing them through a correlogram that is invariant to the changes in scale. The computational complexity of these approaches [14, 16–18] increases with the increase in the size of codebook [11].

The second approach to compute the spatial information is based on the division of image into geometric sub-regions such as rectangles [10], triangles [11, 13] and circles [12]. The most notable research for this domain is Spatial Pyramid Matching (SPM) [10] that sub-divides an images into several rectangular cells. A weighted pyramid-based scheme is applied for the computation of histogram of visual words from each of the divided cell. Inspired from the efficient and effective performance of (SPM) [10], triangular [11, 13] and circular [12] sub-divisions are also applied for the computation of histograms for BoF model to capture the spatial attributes of images. All of these approaches [10–12] represent an image in a large dimensions as compared to standard BoF model as histograms equal to the size of codebook are computed from each of the divided sub-region. The increase in this semantic dimensions of resultant histogram is beneficial, as it captures image spatial information that is also useful for the learning of classification-based model [10–12].

**Fig 1:**
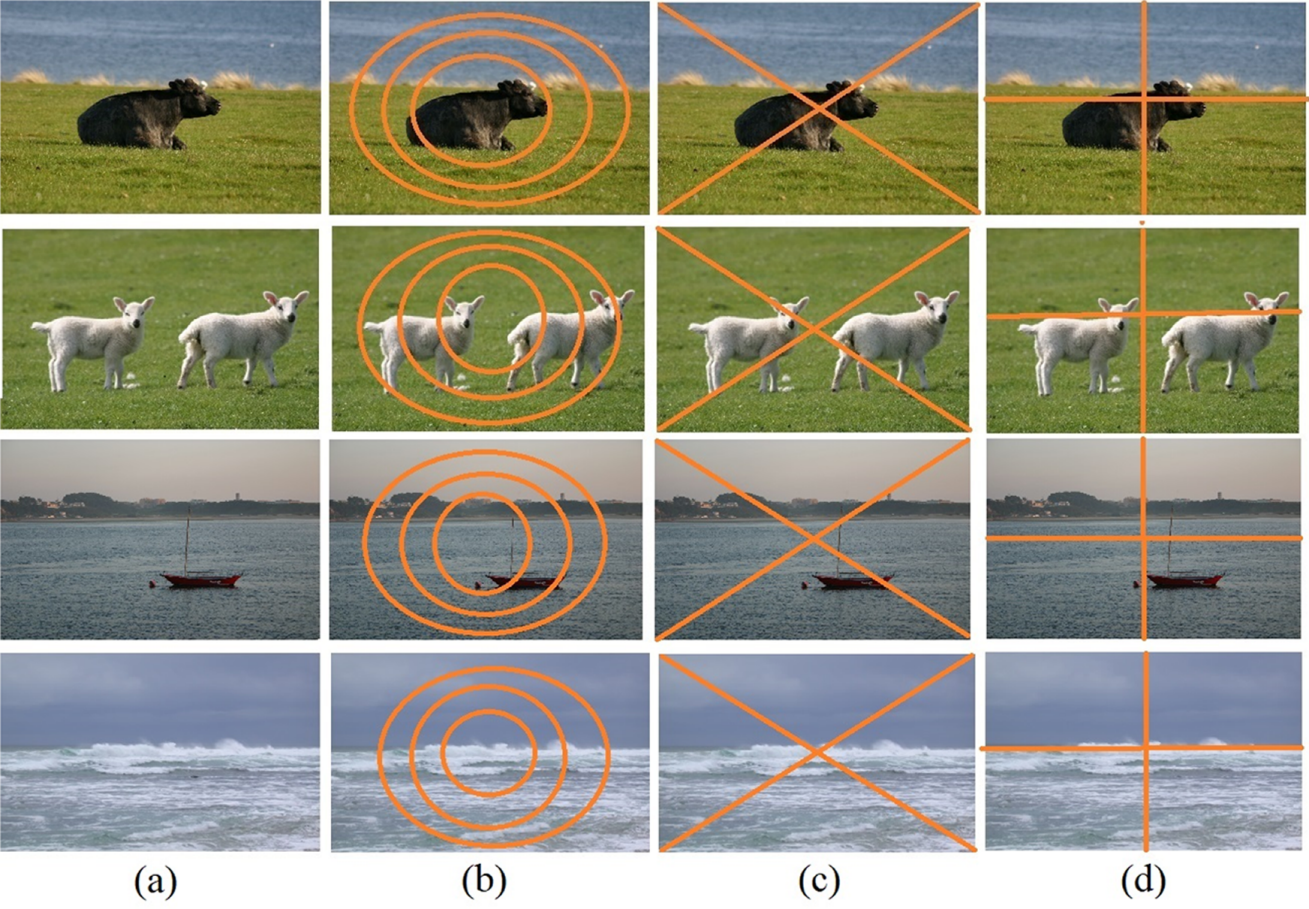
Images taken from the different classes of MSRC-v2 image database [18].

Images of a dataset may contain various transformations such as changes in scale, position of object at different locations and multiple objects in the same scene. Fig 1 represents the images taken from different semantic classes of the MSRC-v2 image database [18]. The images shown in first to fourth row belong to the semantic classes “cow, grass”, “sheep, grass” and “water, boat”, respectively (the images shown in the third and fourth row belong to the same semantic class that is “water, boat”). The sub-figures b,c and d for the respective class show the division of image into circles, triangles and rectangles. From Fig 1, it can be seen that in some cases the area or object of interest such as cow lies within the circle for the computation of spatial histograms of visual words. In case of division of image into triangular cells, we can see that the areas or regions of interest such as sky, water and grass are likely to be situated within the top and bottom cells of triangles [11]. In case of rectangular divisions, we can see that animals and ships are divided into various rectangles and the visual words are splitted across respective histograms. In case of standard BoF model, non-spatial histogram is computed form the whole image, while in case of image division into sub-regions, separate histograms are constructed from each of the divided sub-region [10–12]. This technique provides an option to represent an image in a larger dimensions on a smaller size of constructed codebook [10–12]. This is beneficial for image representation as it captures the image spatial attributes that are also beneficial for the learning of classification-based model [10–12]. Here it is important to mentioned that the geometric sub-divisions of image (circular, triangular and rectangular) are different from image segmentation, as it divides the image at the time of computation of histogram by following a fixed rule (circular, triangular or rectangular). The main contribution of this paper is to propose a novel image representation that is based on a Hybrid Geometric Spatial Image Representation (HGSIR). Each image is divided into circles, triangles and rectangles and histograms of visual words are constructed from each of the divided region. Later on, all the constructed histograms for a single image are concatenated to represent the image in the form of a histogram based on HGSIR.

The structure this research article is as follow: section 2 is about literature review and related work. Section 3 is about BoF model and is about the proposed methodology that is based on computation of spatial information. Section 4 is about image datasets, experimental parameters, results and discussion, while section 5 is about conclusion and future directions of research.

## 2 Related work

In recent few years, there is an increase in multimedia contents and digital images play a major role in various applied applications such as remote sensing, medical care, scene analysis, forestry and image retrieval [19–23]. The basic requirement for image classification is to assign the labels to the images so that they can be arranged in any of the pre-defined category [16]. The performance for any image classification-based system depends on the training of classifier. In BoF model, the final feature vector is the order-less histogram of visual words that is used as an input for the training of classifier [16]. The representation of image spatial attributes in the histogram for BoF model has shown good results in various image classification-based problems [16]. Researchers have proposed different image representations to address the problem for the BoF based image representation. The first group is based on visual words co-occurrences/ geometric relationships such as angle and distance among visual words [14, 16, 17], while the second group sub-divides the image into geometric regions and histograms for BoF model are computed over the divided sub-regions [10–12].

Khan et al. [14] captured the global spatial attributes of images by computing the angle histogram among PIWs. The proposed angle histogram-based image representation captured the global spatial attributes that are reported invariant to transformations such as translation and scaling but suffers in case of image rotations. To deal with image rotations, Anwar et al. [17] computed the triplets within the circular regions of image and evaluated triplets for ancient coins datasets. Later on, Zafar et al. [16] extended the previous work [14, 17] by computing an orthogonal vector for triplets of identical visual words. The final histogram-based representation is computed by using magnitude of these orthogonal vectors. The approaches discussed above [14, 16, 17], are based on the geometric relationships among visual words and computational complexity of these approaches increases exponentially with the increase in the size of codebook [14, 17].

**Fig 2:**
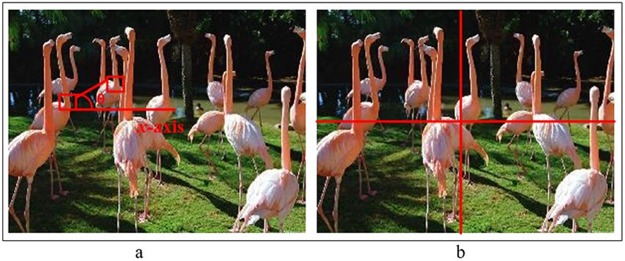
Fig (a) shows the approach based on geometric relationships among visual words [14] (b) SPM approach based on histograms of geometric sub-regions (rectangular) [10].

Lazebnik et al. [10] proposed SPM and captured the spatial attributes of image to enhance the classification accuracy of BoF model. The image is sub-divided into rectangular regions of different sizes and histograms of visual word are computed over each sub-divided rectangular region. The final feature vector for BoF-model is computed by applying a weighted scheme on three different levels and image is represented in a higher-dimensional feature space as compared to the standard BoF model [24]. Fig 2 provides an illustration of the PIWAH (visual words co-occurrences/ geometric relationships) [14] and SPM (image sub-divisions) [10] approaches. Inspired from the concept of SPM, Ali et al. [11] computed the image spatial attributes by dividing an image into different triangular cells and presented an idea about the histograms of triangles (level-1 and level-2). For level-1 triangles, the dimension of resultant histogram is twice the size of constructed codebook, while for level-2 triangles the size of feature vector is four times the constructed codebook [11]. Li et al. [25] computed the image spatial attributes by using Spatial Pyramid Ring (SPR) for scene classification-based problem. The SPR is reported rotation invariant [25] as circular regions are used for histogram computation.

According to Piotr et al. [26], the geometric sub-divisions for the computation of spatial clues are applied in many recent object recognition and image classification techniques, as this can provide coarse-to-fine spatial attributes. Inspired for this idea [10], the spatial information among local descriptors is computed by using Spatial Coordinate Coding (SSC) with semi-coding. The initial spatial component is computed at the local descriptor-level while the other is computed through SPM [10]. The experimental results and analysis stated that pyramid matching can be applied with color and dominant angle [26]. Krapac et al. [27] applied a Fisher kernel framework based on Gaussian Mixture Model (GMM) with soft-assignments to encode the image spatial attributes by using spatial pyramid representation. The image spatial layout is combined with Fisher kernel to compute the appearance of local features. The results and comparisons stated that the use of Fisher kernel with image spatial layout and soft assignments is computationally efficient with linear classifiers [27]. According to SáNchez et al. [28], the computation of averaging local-statistics features for BoF model can enhance the performance of image classification. The image spatial layout is computed through the representations that are based on average statistics. The experimental results and comparisons stated that the traditional ways to capture the image spatial layout based on spatial pyramid increase variance and reduced variations. To address this problem, the two different approaches are proposed that can balance the two features that are variance and variations [27].

In addition to the computation of spatial information, there are other approaches that can be used to enhance the performance of image classification [1]. Feature fusion [1] is considered as one of the technique that can enhance the performance of image classification and object recognition. The type of feature, either local or global contains the discriminating visual information in the form of feature vector [29]. The global features are applied to represent the entire image, while local feature are used to represent the information about image patches [29]. Kabbai et al. [1] proposed a hybrid visual descriptor for BoF model to represent an image in the form of color and texture. For computation of global features, the authors [1] applied wavelet transform with a modified version of local ternary pattern while Speeded-Up Robust Features (SURF) are used for the computation of local information among image patches. All the visual features (both local and global) are computed by using three color planes [1]. According to Xie et al. [30], the BoF model for image classification treats the visual features as nouns and this ignores useful information. The authors suggested [30] to treat the image visual features as adjectives and proposed a framework to combine the adjectives based on color, shape and image spatial attributes. The experimental results are conducted by using various scene-based image dataset and adjective-based approach is reported superior in terms of classification accuracy with reasonable computational cost [30].

The approaches that are discussed above are based on traditional feature extraction and machine learning techniques [1, 14, 16, 17, 26–30]. The recent research for image classification and machine learning-based problems is shifted to the use of Deep Convolutional Neural Networks (DCNNs) [31–35]. Cheng et al. [33] stated that the use of convolution features can enhance the image classification accuracy of BoF model and proposed Bag of Convolutional Features (BoCF). The research of Cheng et al. [33] is different from the traditional approaches as the visual words are not based on handcrafted features and convolutional neural network is applied to compute the deep convolutional features. The application of BoCF [33] enhances the effectiveness in terms of classification accuracy for scene analysis. According to Scott et al. [34], CNNs are suitable for large-scale image classification models with sufficient training samples. The performance of CNNs is evaluated by using satellite images in Transfer Learning (TL) mode to obtain fine-tuning for the classification of satellite images. TL is selected as it allows to boost the performance of a DCNNs by preserving the previous features extracted over a different domain of images. In another research [35], the fusion technique is applied to combine multiple DCNNs by placing the main focus at the classification. The approaches based on the use of DCNNs obtained higher classifier accuracy with a higher computational cost [35]. Here it is important to mention that the image representation approach presented in this paper is simple, robust and it provides a comparable performance with low computational cost as compared to the recent approaches based on DCNNs [33–35]. On the basis of classification accuracy and other comparisons that are conducted in this paper, it can be stated that the proposed research demonstrates an effective performance and can be applied in a domain for scene analysis and image classification. It can be concluded that the proposed HGSIR provides an effective image classification performance with the advantage of scalability.

## 3 Proposed research

The proposed research is based on the late fusion of visual words that are constructed through different geometric regions of image. Each image is divided into rectangles [10], triangles [11], circles [12] and histograms of visual words are constructed for each of the divided region. Later on, all the constructed histograms for a single image are concatenated to represent the image in the form of a histogram based on HGSIR.

Each approach i.e. circles, rectangles and triangles, has its strengths and limitations. The simplicity and efficiency of rectangular method, in combination with its tendency to yield unexpectedly high recognition rates on challenging data, makes it a good base-line for calibrating new datasets and for evaluating more sophisticated recognition approaches [10].

Semantic information is available at the top, right, left and bottom of the image. Discriminating objects and regions of interest are usually located in different sub-regions of the image. The construction of histograms from triangular regions of the image reduces the semantic gap and adds discriminating information to image representation, in the form of objects and regions of interest that are located at the top, left, right and bottom of the image. The triangles approach has been applied for image retrieval [11].

The standard BoVW model lacks spatial information and the approaches based on the division of images into cells to create histograms of visual words do not allow rotations and changes in view-point. The circular approach constructs the histograms of visual words by dividing images into circular regions and can handle the changes in view point, rotations and computation of spatial information [12]. We have combined the above said three approaches for image representation to enhance the classification accuracy of BoF model.

**Fig 3:**
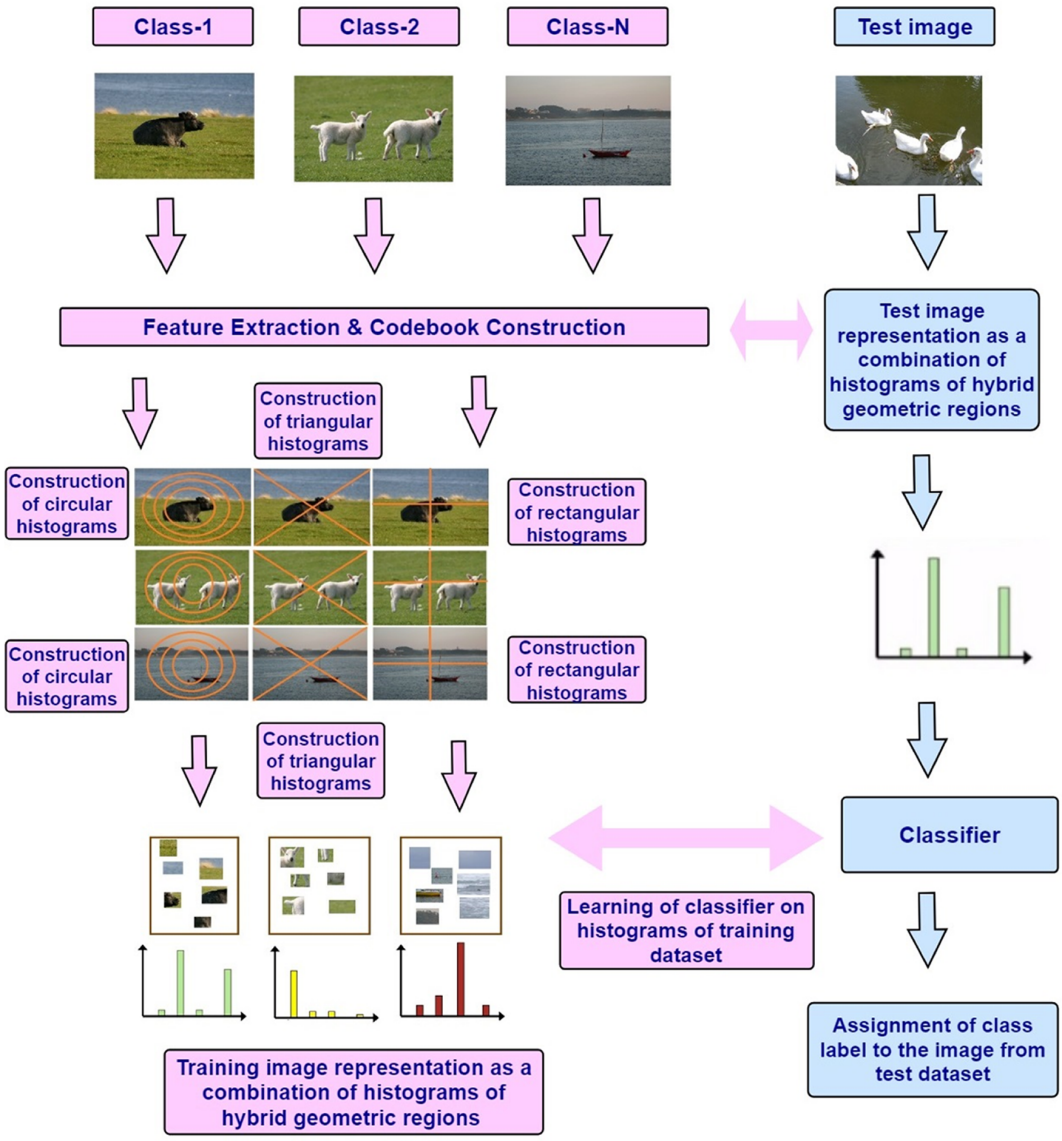
The block diagram of proposed research based on HGSIR.

The block diagram of proposed framework is shown in Fig 3. The BoF model [9] is used to evaluate the performance of proposed research, the detail about the construction of histograms for the proposed HGSIR is mentioned in the following sub-section.

### 3.1 Proposed Hybrid Geometric Spatial Image Representation

1. In BoF model, a two dimensional image with name IMG is represented as:
  $$\begin{array}{c} IMG=I_{\;(m,n)} \end{array}$$ (1)
  where *I*_*m,n*_ are the coordinates or pixels at the spatial location *(m,n)*.
2. Interest point detectors are applied to compute the local features and resultantly the IMG can be expressed mathematically as:
  $$\begin{array}{c} IMG=\{LFD_{1},LFD_{2},\ldots .LFD_{M}\} \end{array}$$ (2)
  Where *LFD_1_* to *LFD_M_* are the descriptors that are computed along the detected interest points.
3. The local features are in a high-dimensional space, therefore feature space is reduced through a quantization algorithm such as *K*-means. The aim of *K*-means is to compute a visual dictionary or a codebook with *N* clusters. We selected *K*-means for quantization due to the its simple and efficient implementation as compared to other clustering approaches such as hierarchical clustering [36]. The codebook *CB* with *N* numbers of clusters is represented as:
  $$\begin{array}{c} CB=\{C_{1},C_{2},\ldots .,C_{N}\} \end{array}$$ (3)
  where *C_1_* to *C_N_* are the constructed clusters.
4. To add the spatial information from circular regions, histograms of concentric circles are created [12]. The partitioning of image into regions at each level is done in a concentric circles fashion, where the *l*^*th*^ level has *l* + 1 regions. Each extracted region is then represented by a histogram of visual words. For an image *IMG* of size *R* × *C*, the centroid *c* = (*c*_*x*_, *c*_*y*_) of an image is calculated as
  $$\begin{array}{c} c_{x}=\frac{1}{|IMG|}\sum_{i=1}^{|IMG|}x_{i},\;\;c_{y}=\frac{1}{|IMG|}\sum_{i=1}^{|IMG|}y_{i} \end{array}$$ (4)
  where *IMG* = {(*x*_*i*_, *y*_*i*_) ∣ 1 ≤ *x*_*i*_ ≤ *C*, 1 ≤ *y*_*i*_ ≤ *R*} and ∣ *IMG* ∣ is the number of elements in *IMG*. Let *L* be the number of levels, then the radius *r* of *l*^*th*^ level is given by
  $$\begin{array}{c} r_{l}=\frac{l}{L}\;min\left\{c_{x},c_{y}\right\} \end{array}$$ (5)
  The radius of the smallest circle will be *r*_1_.
5. To map the visual words on the circular regions, the nearest clusters are assigned to the quantized features by using the following equation:
  $$\begin{array}{c} C\left(LFD_{k}\right)=\underset{C\;\varepsilon \;CB}{\text{argmin}}\;Dist\left(C,LFD_{k}\right) \end{array}$$ (6)
  where *C(LFD_k_)* is representing the cluster (visual word) that is assigned to the *k^th^* feature *LFD_k_* while *Dist(C,LFD*_*k*_*)* shows the distance of computed feature *LFD_k_* and the cluster center *C*. Each patch of image is represented in the form of visual words.
6. Consider *E_i_* is the group of all features that are assigned to the cluster *C_i_*, then the *i^th^* bin of the histogram of visual words *b_i_*, is the cardinality of the set *E_i_*.
  $$\begin{array}{c} b_{i}=Card\left(E_{i}\right)\;and\;E_{i}=\left\{LFD_{k},k\in \left(1,\ldots .,M\right)|C\left(LFD_{k}\right)=C_{i}\right\} \end{array}$$ (7)
7. The spatial histograms computed over the circular regions of image are mathematically expressed as:
  $$\begin{array}{c} Hist_{Cir}=\left\{hist_{{}_{C}R1},hist_{{}_{C}R2},\ldots .hist_{{}_{C}RN}\right\} \end{array}$$ (8)
  where *Hist_Cir_* are the circular spatial histograms and *hist_CR1_* to *hist_CRN_* are the number of divided circles and dimension of visual words computed through each histogram over a circular region is equal to the size of constructed codebook.
8. The histograms of visual words for level-2 triangles [11] based on image triangular sub-divisions are computed and step number 5-6 are repeated. The resultant histograms of triangles are mathematically expressed as:
  $$\begin{array}{c} Hist_{Tri}=\left\{hist_{{}_{T}R1},hist_{{}_{T}R2},\ldots .hist_{{}_{T}RN}\right\} \end{array}$$ (9)
  where *Hist_Tri_* are the triangular spatial histograms and *hist_TR1_* to *hist_TRN_* are the number of divided triangles and dimension of visual words computed through each histogram over a triangular region is equal to the size of constructed codebook.
9. The histograms of visual words for level-1 rectangles [10] based on image rectangular sub-divisions are computed and step number 5-6 are repeated. The resultant histograms of rectangles are mathematically expressed as:
  $$\begin{array}{c} Hist_{Rect}=\left\{hist_{{}_{R}R1},hist_{{}_{R}R2},\ldots .hist_{{}_{R}RN}\right\} \end{array}$$ (10)
  where *hist_Rect_* are the rectangular spatial histograms and *hist_RR1_* to *hist_RRN_* are the number of divided rectangles and dimension of visual words computed through each histogram over a rectangular region is equal to the size of constructed codebook.
10. In the last step, the histograms of visual words that are computed using circular, triangular and rectangular geometric regions are vertically concatenated to represent image in the form of histogram of hybrid geometric regions. The final feature vector that is the histogram of visual words of hybrid geometric regions is expressed as:
  $$\begin{array}{c} HGSIR=\{Hist_{Cir};Hist_{Tri};Hist_{Rect}\} \end{array}$$ (11)
  where *HGSIR* is the final spatial histogram based on visual words computed over hybrid geometric regions of image.

## 4 Experimental datasets and results

This section is about the selected image datasets, implementation details, image classification and results obtained form the proposed research. We selected 15-scene image benchmark for the evaluation of proposed research that contains fifteen semantic classes. It is the most widely used dataset for the evaluation of research for image classification and object recognition. This dataset contains a wide range of in-door and out-door images, there are total of 4485 images (200-400 images per semantic class) with an average size of 300 × 250 pixels. The photo gallery of the images taken from the 15-scene dataset is shown in Fig 4.

**Fig 4:**
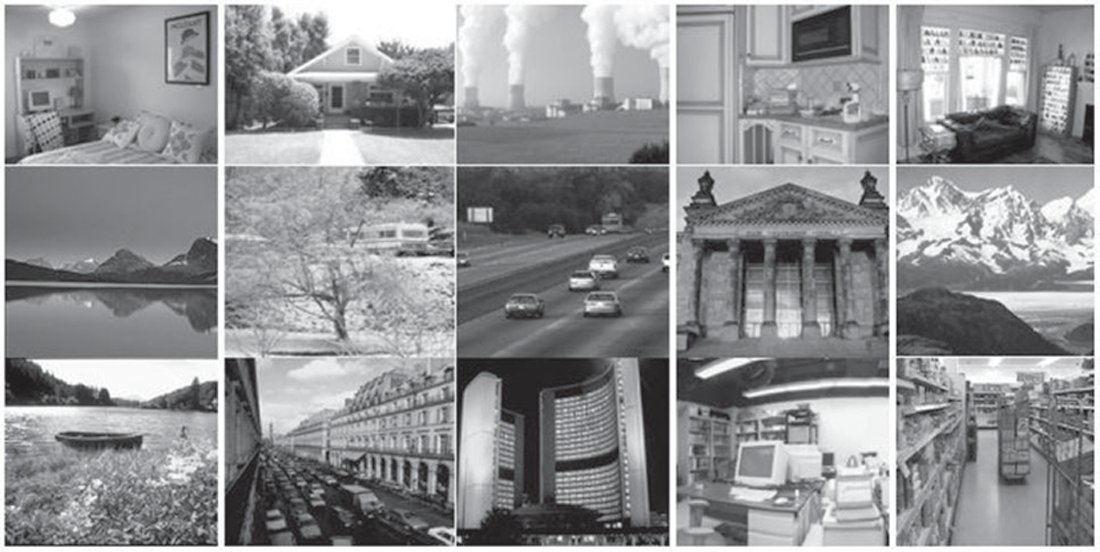
The photo gallery of images representing each class of 15-scene image dataset.

The details about the class titles/lables and number of images per class is referred to [10, 14]. To perform a fair comparison with the existing research in terms of classification accuracy, we selected 100 images from each of the class of 15-scene image benchmark for training and remaining for testing (1500 training images and 2985 test images). The same percentage of training and testing is being used in the research that is selected for comparison.

**Fig 5:**
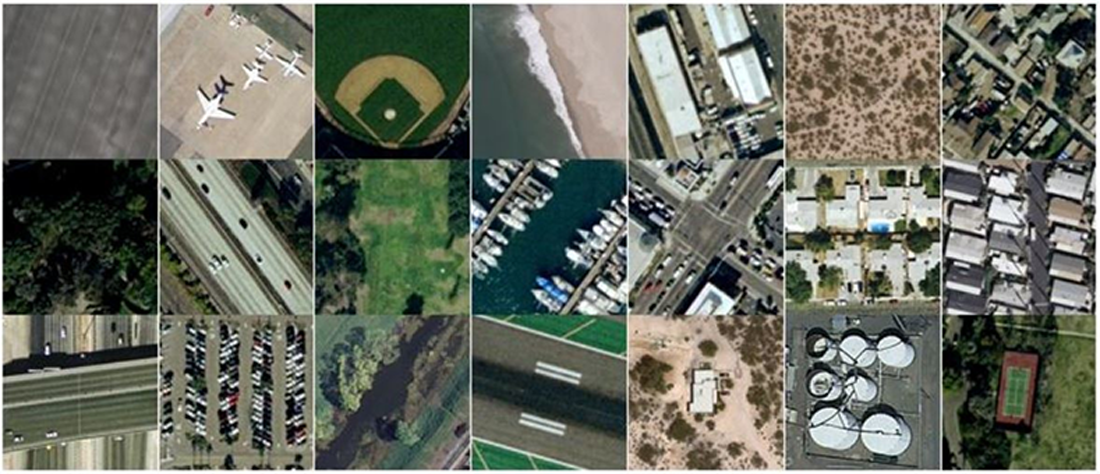
The photo gallery of images representing each class of UCM dataset.

UC Merced (UCM) Land Use image dataset is also selected to evaluate the performance of the proposed research. This dataset was created by Yang et al. [37] and it contains 21 classes, with a uniform distribution of 100 images per class. The photo gallery of images from UCM dataset is shown in Fig 5.

The details about the class titles/lables and number of images per class is referred to [37]. We followed the experimental setup as mentioned in [37–39], by a random selection of 80 images for each class for training and the remaining for testing, with a training-testing ratio of 1680-420 images respectively.

**Fig 6:**
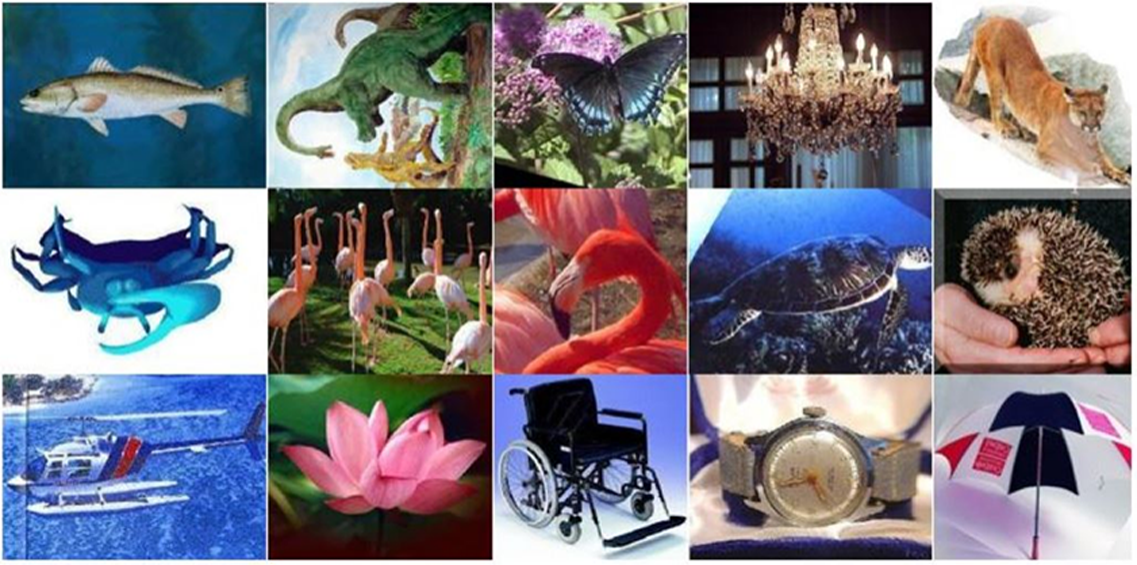
The photo gallery of images selected from the Caltech-101 dataset.

The third dataset is the Caltech-101 [40], that was created in 2003 and there are 101 object categories in this dataset (animals, furniture, vehicles etc) with a total of 9144 images. There are 40-800 images per class with an average image size of 300 × 200 pixels. For the sake of comparisons, the dataset is randomly divided by using a training-testing ratio of 0.6:0.4. The photo gallery of images selected from some categories of the Caltech-101 dataset is shown in Fig 6.

**Fig 7:**
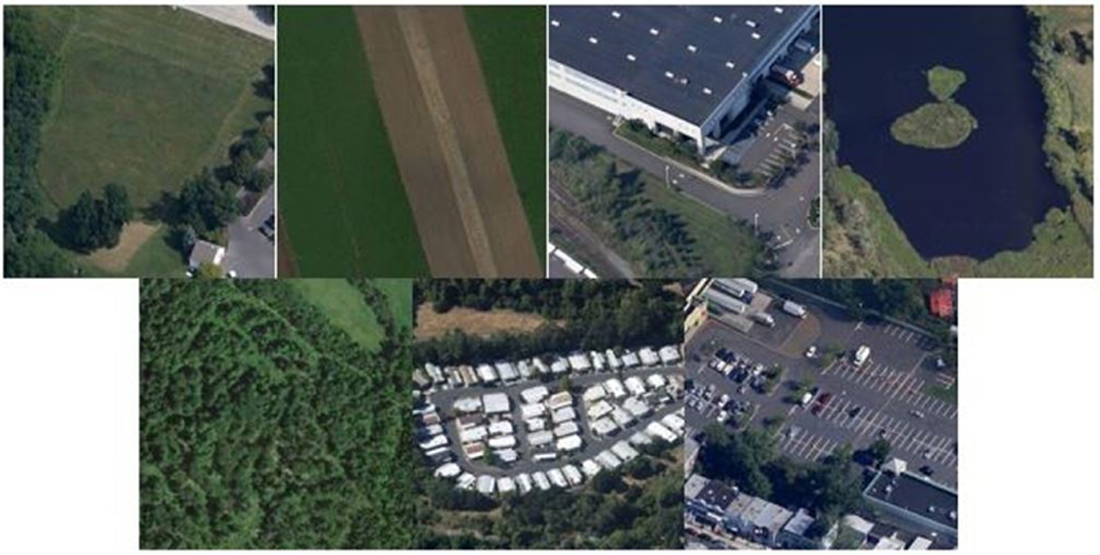
The photo gallery of images selected from the RSSCN7 image dataset.

The forth dataset used to evaluate the performance of proposed image representation is the RSSCN7 dataset [41]. There are total of 2800 images of remote sensing with 07 different classes. The details about the class titles/lables and number of images per class is referred to [41]. To ensure fair comparison, the training-testing ratio for this dataet is 0.5:0.5 is consistence with the related works [42]. The photo gallery of images from this dataset are shown in Fig 7.

**Fig 8:**
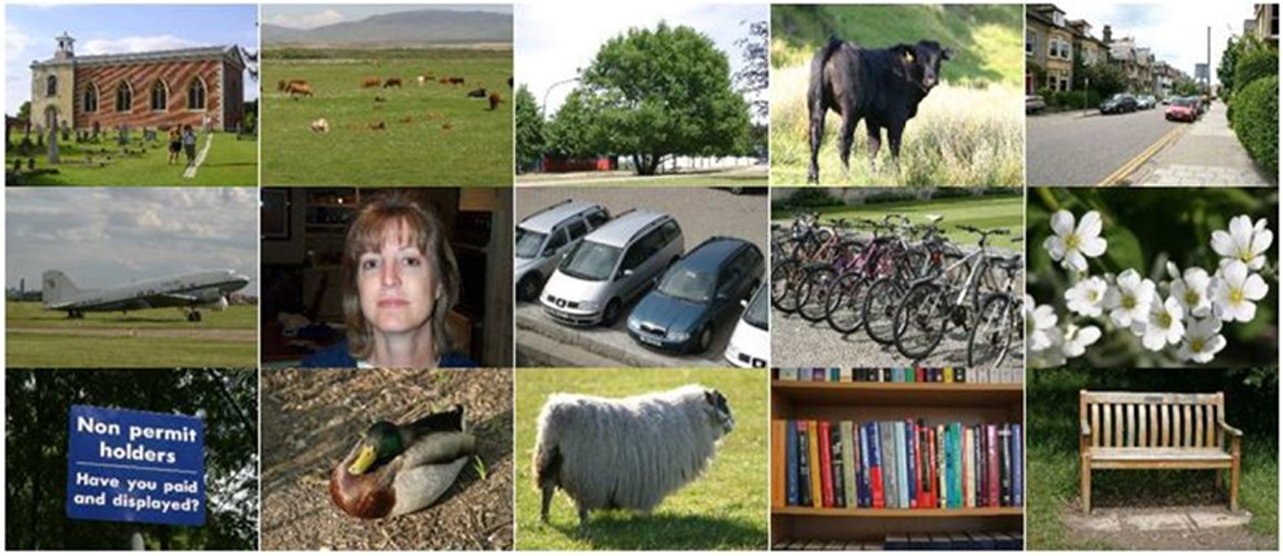
The photo gallery of images selected from the MSRC-v2 image dataset.

Finally, the results are also collected for the MSRC-v2 image dataset. It consists of 591 images classified into 23 different categories. The details about the class titles/lables and number of images per class is referred to [14, 18]. The training and testing sets are randomly selected using a training-testing ratio 0.6:0.4. The photo gallery of images from MSRC-v2 dataset is shown in Fig 8.

### 4.1 Implementation details

For all datasets, the image representations are created by following the same experimental steps. We repeated every experiment 10 times with different realizations of training and test images to reduce the influence of randomness. As a pre-processing step, all the images are converted to gray-scale to extract dense SIFT features with a dense grid of size 8 and computed SIFT descriptor after evert 8th pixel. To quantize these descriptors, *K*-means clustering is applied and computational cost of clustering is reduced by selecting 0.5% of random features from the training dataset (for codebook computation) [43]. The size of visual vocabulary is an important parameter that has a significant impact on the classification accuracy. The performance is directly proportional to vocabulary size, while a larger vocabulary size tends to over-fit [43]. The experiments are performed with different sizes of vocabulary to sort out the best performance obtained from the proposed research. Since our approach adds spatial information after visual vocabulary construction, the images are then partitioned into regions according to different schemes to obtain the spatial histograms. The histograms constructed from different levels are concatenated to create the histogram representation for each relevant scheme. The spatial histograms are then normalized. The final hybrid histogram based representation is obtained by combining the histograms obtained through each scheme.

The dimensions of Rectangular (Rect), Triangular (Tri) and Circular (Cir) histograms are given by

$$\begin{array}{ccc} dim(Hist_{Rect}) & = & K_{Rect}\times R_{Rect}\\ dim(Hist_{Tri}) & = & K_{Tri}\times R_{Tri}\\ dim(Hist_{Cir}) & = & K_{Cir}\times R_{Cir} \end{array}$$

where *K* is the size of visual vocabulary and *R* is the number of regions. As we have partitioned the image upto level-1 for Rect, level-2 for Tri and level-3 for Cir, (*R* is equal to 4 in all cases). The dimensions of final histogram is computed by vertically concatenating the histograms computed over three geometric regions. This can be expressed as:

$$\begin{array}{c} dim\left(HGSIR\right)=dim\left(Hist_{Rect}\right);dim\left(Hist_{Tri}\right);dim\left(Hist_{Cir}\right) \end{array}$$ (12)

Support Vector Machines (SVM) is an example of supervised classification [8], given the + *ve* and −*ve* training images, the objective is to classify a test image whether it contains the object class or not. We applied Hellinger kernel [44] with linear SVM on the normalized histograms of visual words computed through proposed approach. The best value *C*, that is parameter of linear SVM is computed through 10-fold cross validation by using training images. To demonstrate the effectiveness of the proposed approach, we compared the classification accuracy obtained from circular, triangular and rectangular histograms for every image dataset (using the same set of training and test images for the respective iteration).

### 4.2 Classification of 15-scene image dataset

**Fig 9:**
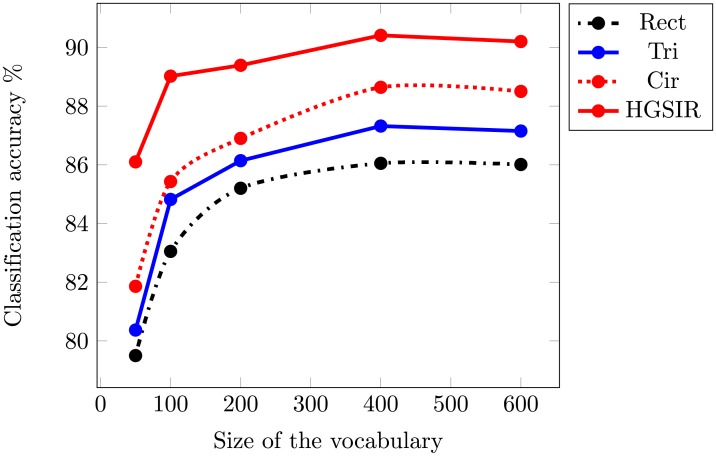
The mean classification accuracy comparison while using different sizes of visual vocabulary for 15-scene image dataset.

To ascertain the optimal performance for accurate feature representation, experiments are performed with visual vocabulary of different sizes. From Table 1, it can be observed that the best performance for HGSIR i.e. 90.41% is obtained for a vocabulary of size 400. For all other approaches, the optimal performance is obtained for the same vocabulary size i.e. 400 (as illustrated in Fig 9 through a plot). The classification accuracy obtained from the proposed HGSIR is higher than the other approaches based on computation of spatial information. Our method provides 4.36% higher accuracy compared to Rect, 3.09% more than Tri and 2.52% higher accuracy compared to the second best method i.e Cir.

**Table 1:** Comparison of classification accuracy while using different sizes of vocabulary.

Voc. Size	Rect	Tri	Cir	HGSIR
50	79.5%	80.37%	81.86%	86.1%
100	83.05%	84.82%	85.43%	89.02%
200	85.2%	86.14%	86.9%	89.39%
400	86.05%	87.32%	87.89%	**90.41%**
600	86.01%	87.15%	87.75%	90.2%

The above comparisons demonstrate the effectiveness of the proposed HGSIR as compared to the state-of-the-art concurrent methods. We also compared HGSIR with the recent methods focused to enhance the classification accuracy using different approaches such as spatial context and feature fusion techniques. It is clearly evident from the Table 2 that the proposed hybrid representation gains the highest classification accuracy.

**Table 2:** Comparison with existing research in-terms of classification accuracy while using 15-scene image dataset.

Algorithms	Accuracy
SPM Entire Pyramid (*L* = 2) [10]	81.4 ±0.5
Zang *et al*. [45]	81.5%
PIWAH+ [14]	82.5%
LVS+SIFT [46]	83.2±0.58%
SPS_ad_+ [15]	83.7%
Karmakarei *et al*. [47]	84.2%
EMFS [48]	85.7%
LGF [38]	85.8%
OVH [16]	87.07%
LVFC-HSF [49]	87.23%
CWCH [12]	88.04%
HGSIR	**90.41%±0.72**

The proposed approach provides 9.01% higher accuracy as compared to SPM pyramid level 2 [10]. Khan *et al*. [14] created an image representation by incorporating the relative spatial context termed as PIWAH, that resulted in a classification accuracy of 76%. They proposed to combine PIWAH with SPM [10] in PIWAH+ and achieved an accuracy of 82.5%. HGSIR image representation results in 7.9% higher accuracy as compared to their work. Further, it should be noted here that the approaches based on computing geometric relationships between visual words are computationally expensive [11]. HGSIR provides superior performance to their work in terms of both classification accuracy and computational complexity, as it incorporates the absolute spatial information. Soft Pairwise Similarity Angle Distance Histogram (SPS_ad_+ [15]) combines angle, distance and absolute spatial information to final histogram representation. HGSIR comparatively provides 6.7% better results with reduced computational complexity.

Karmakar *et al*. [47] enhanced the conventional spatial pyramid method to obtain rotation-invariant image classification by partitioning image into concentric rectangles. The proposed approach used concatenated weighted histograms extracted in a rectangular ring fashion from each region at each level. They reported an accuracy of 84.20% using a vocabulary of size 200 with a feature vector of size 4200. Our proposed HGSIR provides 6.21% higher accuracy compared to their work.

Zou *et al*. [38] proposed LGF, a fusion of local and global features and also considered the spatial context by incorporating SPM in implementation. Our proposed representation attains a performance gain of 4.6% over LGF. Huang *et al*. [46] included the spatial information at descriptor level and achieved 83.2% accuracy. Zang *et al*. [45] proposed a framework that utilizes important and useful information from images to simplify OB (Object Bank) representation. OB combines both semantic and spatial information. HGSIR achieves 8.9% higher classification accuracy as compared to their work. HGSIR provides competitive performance to the recent state-of-the-art methods.

Extended Multi-Feature Spatial Context (EMFS) representation [48] is based on combination of multiple features, and the spatial neighborhood resulting in 85.7% classification accuracy. Lin *et al*. [49] proposed a local visual feature coding based on heterogeneous structure fusion to overcome the limitation of capturing intrinsic invariance in intra-class images or image structure for large variability image classification. Our methods provides 3.18% higher accuracy compared to their approach.

OVH [16] is a relative spatial feature extraction method. It is based on extracting global geometric spatial relationships by computing the magnitude of orthogonal vectors between TIWs. HGSIR yields 3.34% better accuracy compared to OVH. CWCH [12] is a recent approach, focused to incorporate the spatial context by partitioning the images in geometric sub-regions. It works by partitioning the images into circular regions and aggregates the weighted histograms from each sub-region and each level in a pyramid fashion. The proposed hybrid approach, HGSIR, outperforms CWCH by obtaining 2.37% higher accuracy. It can be safely concluded that HGSIR provides better performance compared to the state-of-the-art absolute and relative spatial feature extraction methods.

**Fig 10:**
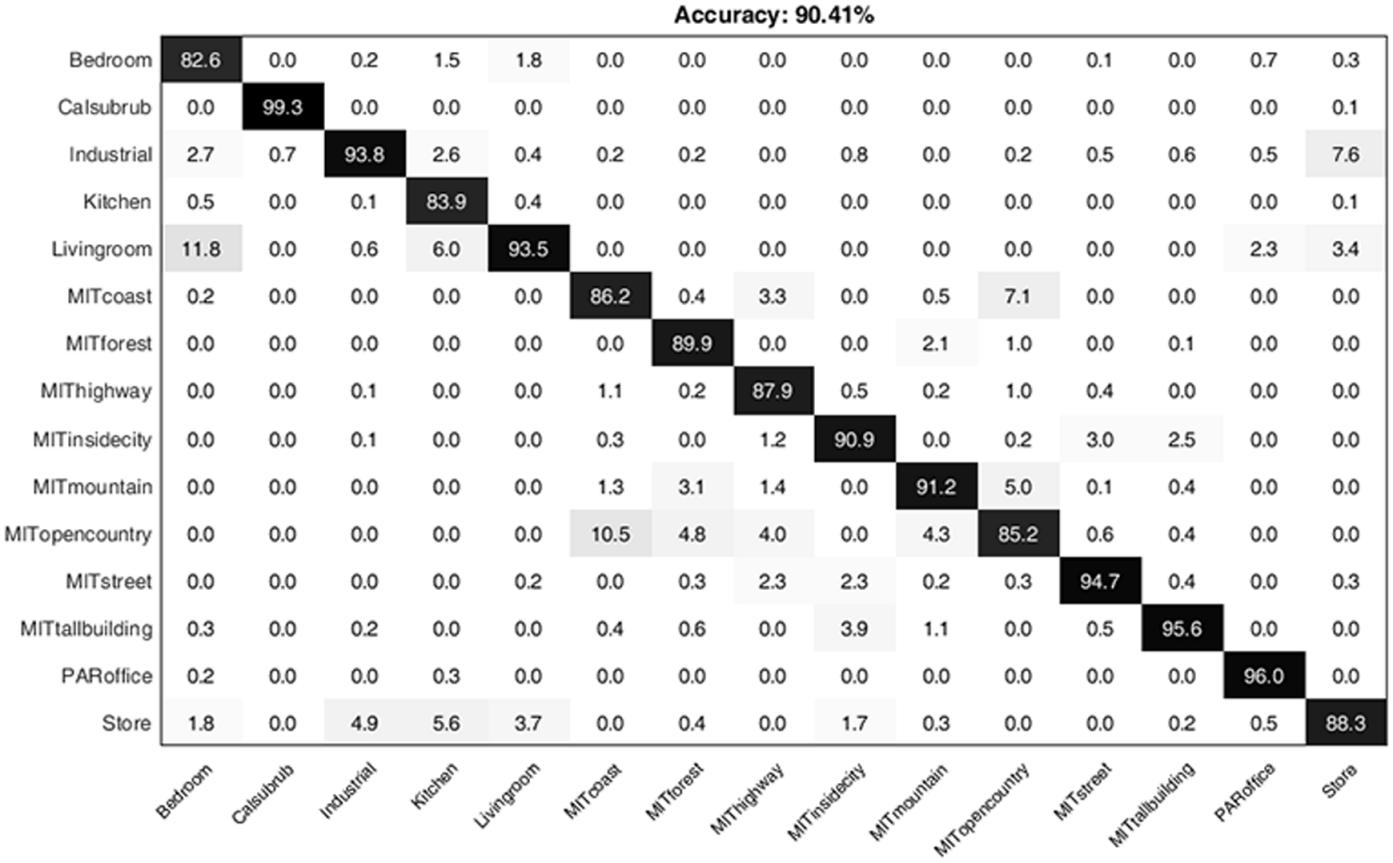
The confusion matrix representing the computed classification accuracy % for the proposed research while using 15-scene image dataset.

The mean confusion matrix for 15-scene image dataset obtained from the proposed research is shown in Fig 10. The diagonal values show the precision normalized percentages for each class.

**Fig 11:**
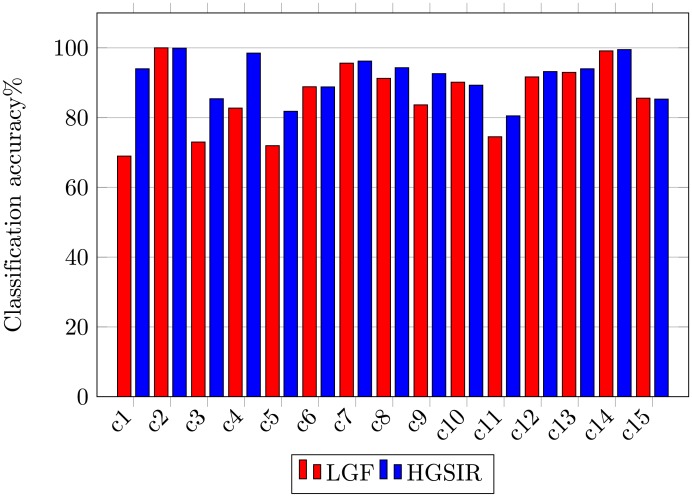
Class-wise comparison between LGF [38] and HGSIR for the 15-scene image dataset.

The class-wise classification accuracy comparison between LGF [38] and the proposed HGSIR is shown in Fig 11. The results show that the proposed research outperforms and provides competitive performance with LGF [38] against all classes for the 15-scene image dataset.

### 4.3 Classification of the UCM image dataset

**Fig 12:**
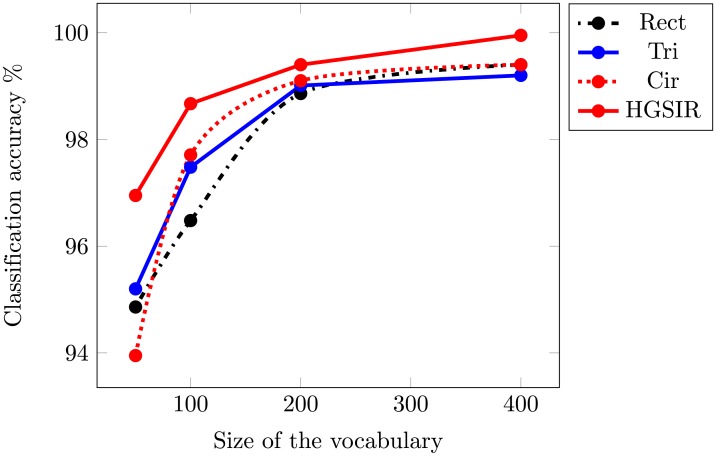
The mean classification accuracy comparison while using different sizes of visual vocabulary for UCM image dataset.

The second dataset used for the evaluation of the proposed research is the UCM image dataset. Fig 12 provides a comparison of the Rect, Tri, Cir and the proposed hybrid approach while using the visual vocabulary of different sizes. For all the approaches, the highest performance is obtained for a vocabulary of size 400. The UCM dataset mostly contains land-use scene images at a large scale, hence the spatial information provides important clues leading to the better discrimination. The experimental results validate the effectiveness of the proposed hybrid approach.

In order to further assess the performance of HGSIR, it is compared with the state-of-the-art methods aimed to enhance the classification performance (as shown in Table 3). Zhao *et al*. [50] proposed CCM-BOVW for describing spatial information and implied multiple features for land use scene classification. Our approach provides 13.31% performance gain as compared to CCM-BOVW. Chen *et al*. [51] proposed MS-CLBP descriptor to characterize dominant texture features of multi-resolution images. HGSIR achieves a performance gain of 9.35% over MS-CLBP.

**Table 3:** Comparison with existing research in-terms of classification accuracy while using UCM image dataset.

Algorithms	Accuracy
CCM-BOVW [50]	86.64% ± 0.81%
MS-CLBP_1_ [51]	90.6% ± 1.4%
SOS [52]	94.33%
LGF [38]	95.48%
salM^3^LBP-CLM [39]	95.75% ± 0.80%
LGFBOVW [53]	96.88% ± 1.32%
ResNet50 [34]	98.5%
Zeng *et al*. [42]	99±0.35%
Evolved Sugeno [35]	99.33%
CWCH [12]	99.4%
HGSIR	**99.95%±0.1**

**Fig 13:**
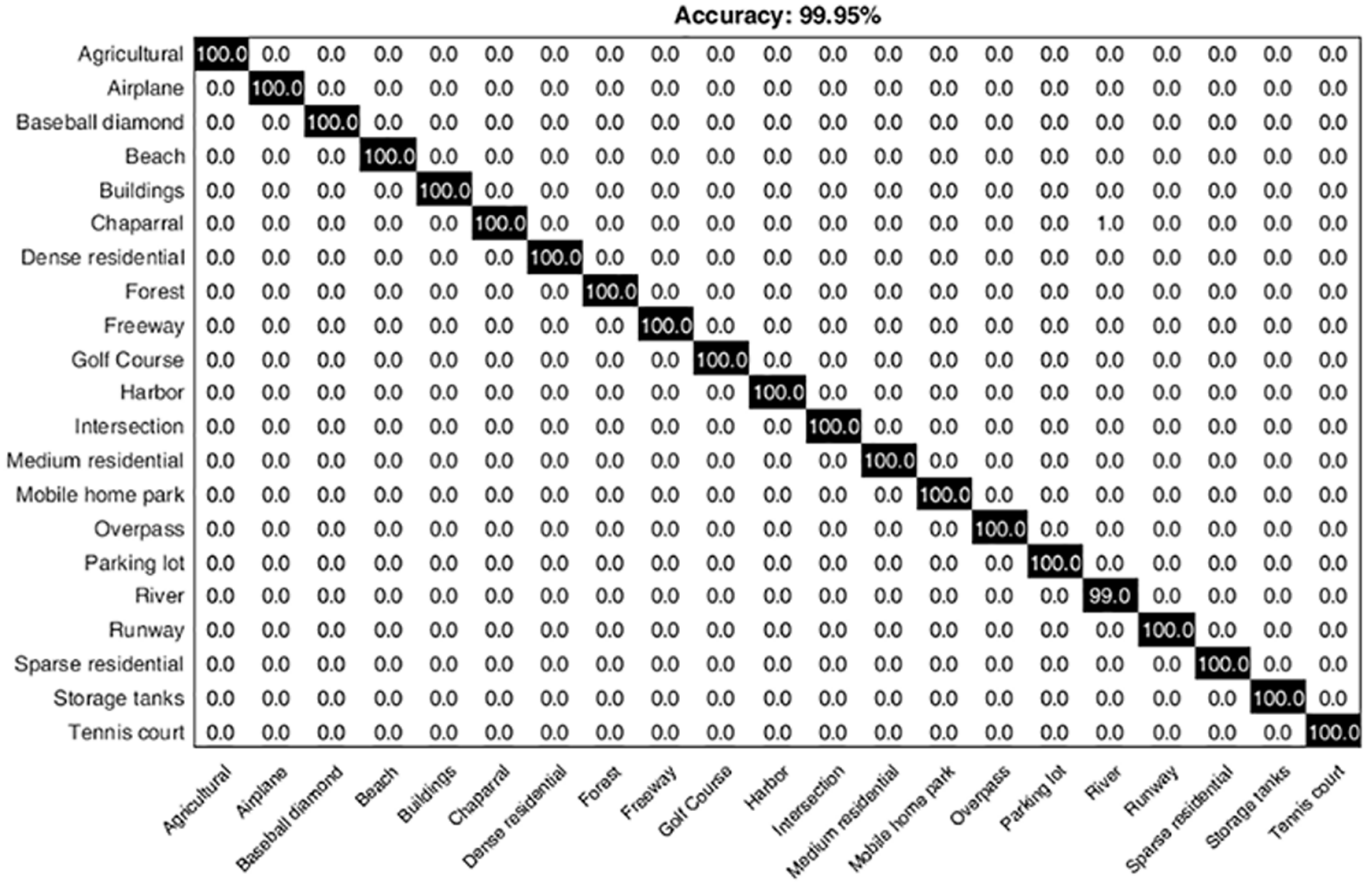
The confusion matrix representing the computed classification accuracy % for the proposed research while using UCM image dataset.

The proposed hybrid approach attains a substantial performance gain over the recent state-of-the-art methods. HGSIR achieves 0.62% highest accuracy as compared to Evolved Sugeno [35], that is based on deep learning. To the best of our knowledge, Scott et al. [35] reported the highest classification accuracy i.e. 99.33% for UCM image dataset using deep learning approaches. Prior to their work, Penatti [54] reported highest classification accuracy that is 99.43% by combining CaffeNet with OverFeat and the outputs were fed into SVM. CWCH [12] is a complementary approach to HGSIR as it is based on spatial feature extraction by using concentric weighted circles, resulting in an accuracy of 99.4%. The proposed approach yields 0.55% higher accuracy compared to CWCH. The proposed hybrid image representation provides competitive performance as compared to the state-of-the-art methods. The confusion matrix for the UCM image dataset is shown in Fig 13. The diagonal values show the precision normalized percentages for each class.

**Fig 14:**
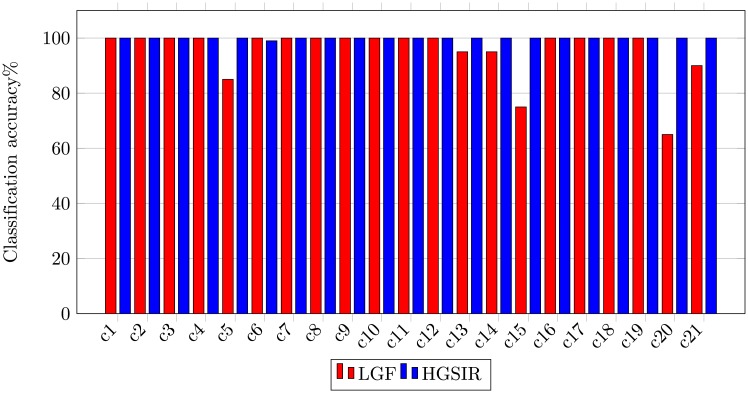
Class-wise comparison between LGF [38] and HGSIR for UCM image dataset.

The class-wise comparison between LGF and UCM image dataset is shown in Fig 14. It can be seen that our method provides major improvement in accuracy of classes i.e. buildings, overpass, storage tanks and tennis court. Significant improvement is also observed in classes medium-residential and mobile home park. Our method provides remarkable results for high resolution scene classification.

### 4.4 Classification of Caltech-101 image dataset

**Fig 15:**
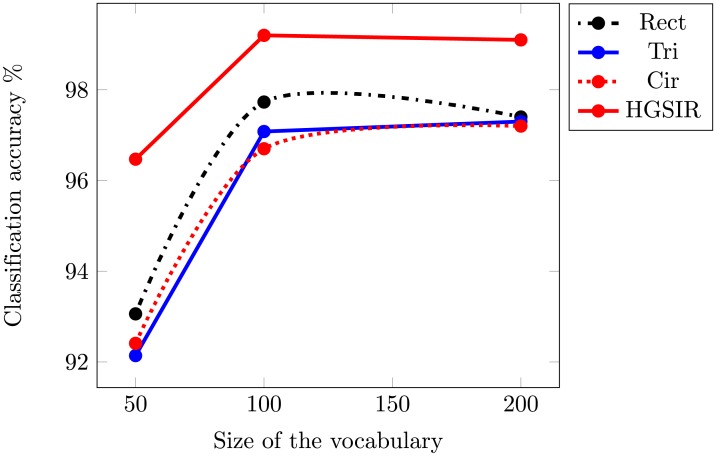
The mean classification accuracy comparison while using different sizes of visual vocabulary for Caltech-101 image dataset.

To further investigate the classification performance of HGSIR, experiments are performed on the challenging Caltech-101 image dataset. Table 4 demonstrates the accuracy attained for the complementary Rect, Tri, Cir and HGSIR approaches over visual vocabulary of different sizes. The optimal performance for HGSIR i.e. 99.2% is obtained for a vocabulary of size 100. Fig 15 provides a graphical comparison between state-of-the-art approaches as a function of vocabulary size.

**Table 4:** Comparison in-term of classification accuracy while using Caltech-101 image dataset.

Voc. Size	Rect	Tri	Cir	HGSIR
50	93.06%	92.14%	92.41%	96.47%
100	97.73%	97.08%	96.7%	**99.2%**
200	97.4%	97.3%	97.2%	99.1%

Table 5 provides a comparison of HGSIR with more recent methods enhancing classification accuracy for the Caltech-101 image dataset by relative spatial information, encoding spatial information at descriptor level and deep learning approaches. Our proposed method provides a performance gain of 34.6% compared to SPM [10], 32.1% compared to PIWAH+ [14], 30.8% as compared to SPS_*ad*+_ [15], 24.2% compared to the LVS+ SIFT [46] descriptor and 20.47 compared LVFC-HSF [49] feature encoding method.

**Table 5:** Comparison with existing research in-terms of classification accuracy for Caltech-101 image dataset.

Algorithms	Accuracy
SPM Entire Pyramid (*L* = 2) [10]	64.6±0.8%
PIWAH+ [14]	67.1%
SPS_*ad*_+ [15]	68.4%
LVS+SIFT [46]	75±0.67%
LVFC-HSF [49]	78.73%
DeCAF_6_ [55]	86.91±0.7%
SVM(VGGI9)+SRSL [56]	92.59%
HGSIR	99.2%

HGSIR achieves 12.29% performance gain over DeCAF_6_ [55] which is based of features extracted from DCNN activation. SVM(VGGI9)+ SRSL [56] in aimed to increase the classification performance by improving feature learning. The proposed approach provides 6.61% higher classification accuracy to the second best reference method. The comparisons demonstrate that the spatial information provides significant clues by enhancing the discriminative power of features.

### 4.5 Classification of RSSCN7 image dataset

**Fig 16:**
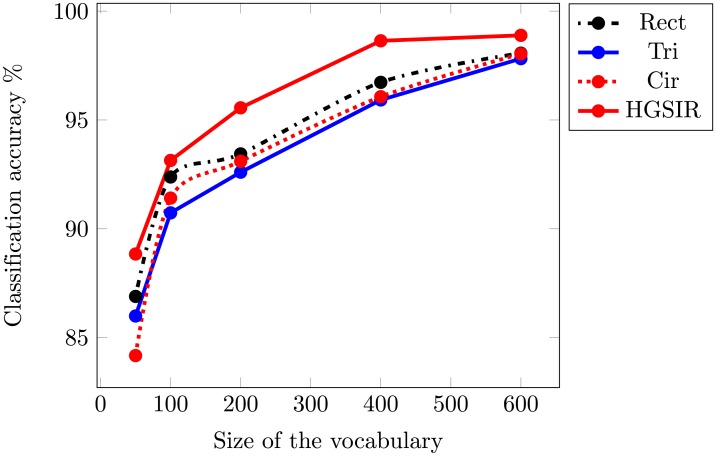
The mean classification accuracy comparison while using different sizes of visual vocabulary for RSSCN7 image dataset.

The RRSCN7 image dataset is a challenging dataset as the images are taken at four different scales and angles. Table 6 provides a comparison of classification performance of Rect, Tri and Cir methods with HGSIR. Our method yields best performance resulting in an accuracy of 98.89%. Fig 16 illustrates the classification performance comparison of these methods over different sizes of visual vocabulary. Our method provides 0.82% higher accuracy to the second best method in comparison. The proposed approach consistently produces remarkable results compared to related approaches.

**Table 6:** Mean average classification accuracy as a function of vocabulary size.

Voc. Size	Rect	Tri	Cir	HGSIR
50	86.89%	85.99%	84.17%	88.84%
100	92.38%	90.73%	91.41%	93.14%
200	93.44%	92.6%	93.1%	95.56%
400	96.73%	95.92%	96.07%	98.64%
600	98.07%	97.82%	98.04%	**98.89%**

Table 7 provides a comparison of the proposed method with recent state-of-the-art approaches. Recently, a research trend is seen to shift to the implementation of deep learning methods for image classification. The deep learning methods have shown outstanding results on most of the datasets. It is worth mentioning here that CNN based methods require huge amounts of data and significant training time to learn the features. Table 7 demonstrates the superiority of the proposed approach to more recent CNN and deep learning based approaches. Zeng et al. [42] applied CNN and improved scene classification by combining global-context and local-object features. The proposed method provides 3.3% higher accuracy compared to the second best method in comparison, despite of the simplicity of the proposed approach.

**Table 7:** Comparison with existing research in-terms of classification accuracy for RSSCN7 image dataset.

Algorithms	Accuracy
VGG16 [57]	87.18±0.94
CaffeNet [57]	88.25±0.62
Deep Filter Banks [58]	90.4±0.6
Anwer *et al*. [59]	94
Zeng [42]	95.59% ± 0.49
HGSIR	98.89%

**Fig 17:**
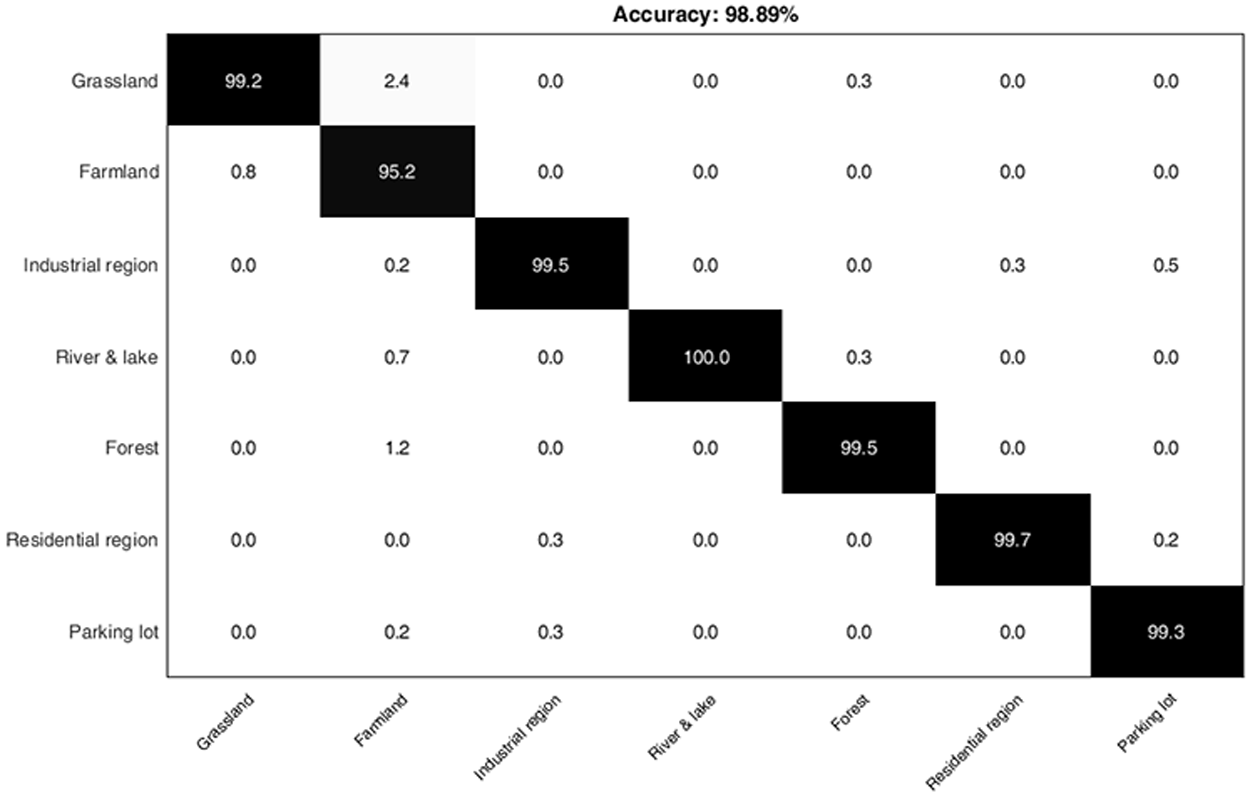
The confusion matrix representing the computed classification accuracy % for the proposed research while using RSSCN7 dataset.

The experimental results demonstrate the efficacy of our approach in recognizing the complex remote scene images. The confusion matrix for the RSSCN image dataset is shown in Fig 17.

### 4.6 Classification of MSRC-v2 image dataset

In order to demonstrate the sustainable performance of the proposed approach, experiments are also conducted by using the MSRC-v2 image dataset. The above comparisons have clearly demonstrated that our proposed HGSIR outperforms the concurrent Rect, Tri and Cir approaches. For MSRC-v2 image dataset, the best performance for HGSIR i.e. 99.89% is obtained for a vocabulary of size 100.

Here in Table 8, we provide a comparison with different state-of-the-art approaches. Savarese *et al*. [18] and Liu *et al*. [60] are the most notable contributions, concerned with modeling geometric relationship between visual words. In addition to this, [60] requires an integrated feature selection and spatial information extraction step. The extraction of spatial information at learning stage would lead to re-computation of features with a modification in training set, hence making it difficult to generalize. Whereas, the approach proposed by Savarese *et al*. [18] requires a 2^*nd*^-order feature quantization step. Despite of the simplicity of the proposed approach, our method provides 18.79% and 16.79% higher accuracy compared to their work. HGSIR yields 17.89% and 16.39% higher accuracies compared to PIWAH [14] and SPS_ad_ [15] respectively. The experimental results validate the robustness of the proposed approach.

**Table 8:** Comparison with existing research in-terms of classification accuracy for MSRC-v2 image dataset.

Algorithms	Accuracy
Savarese *et al*. [18]	81.1%
PIWAH [14]	82.0%
Liu *et al*. [60]	83.1%
SPS_ad_ [15]	83.5%
HGSIR	**99.89%**

**Fig 18:**
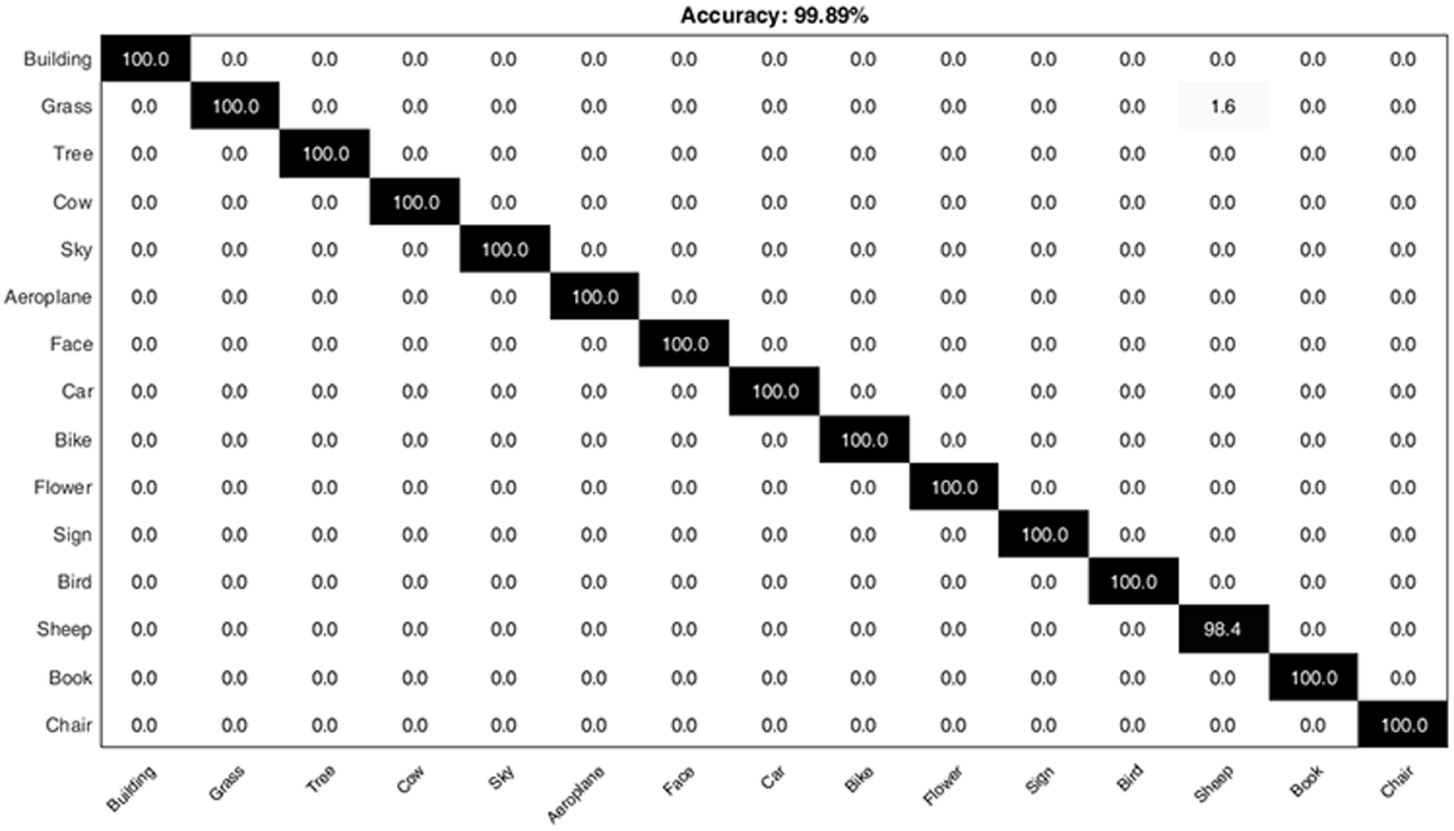
The confusion matrix representing the computed classification accuracy % for the proposed research while using MSRC-v2 image dataset.

The confusion matrix for MSRC-v2 dataset is shown in Fig 18. It can be seen that the only confusion occurs between class Grass and Sheep where some instances of Grass are misclassified in Sheep class. All other classes are correctly classified into their respective semantic categories.

### 4.7 Time complexity

This section is about the training and testing time of the proposed research with complementary approaches. The specifications of the system used to conduct experiments are: Intel(R) Core i7 (seventh generation) 2.70 GHz CPU, 16 GB RAM while using Windows-10 operating system. The proposed algorithms are implemented in MATLAB and the experiments are executed independently each for Rect, Tri, Cir and HGSIR approaches. It is important to mention here that the training time is computed as vocabulary construction + training histograms computation + training of classifier. The testing time is computed as histogram computation of test image and classification using a pre-trained model of classifier. The average CPU time (in seconds) required for HGSIR and the complementary schemes for 15-scene image dataset is presented in Table 9.

**Table 9:** Time comparison for 15-scene image dataset. *K* denotes the size of visual vocabulary.

*K*	Training Time				Testing Time			
	Rect	Tri	Cir	HGSIR	Rect	Tri	Cir	HGSIR
50	285.15	273.47	260.29	652.08	434.46	436.98	434.11	1312.36
100	464.8	484.82	471.85	1283.32	491.32	493.33	490.03	1484
200	1259.718	1419.865	1036.84	2997.223	615.71	621.64	615.34	1867.69
400	1456.66	1691.17	2640.01	3915.19	805.95	814.5	803.77	2440.72
600	3842.55	2946.18	3245.9	5194.86	825.27	827.53	823.58	2476.38

The first observation from Table 9 is that training time increases with the increase in size of visual vocabulary. The increase in the size of visual vocabulary increases the time for the computation of cluster centers and directly impacts the size of resultant feature vector, thereby affecting the overall training time. Same is observed for the testing time, that increases significantly with increase in size of visual vocabulary. The computation time (training and testing) for HGSIR is more compared to the Rect, Tri and Cir approaches owing to the fact, that it involves histogram computation for each of the individual schemes, which are then combined to create the hybrid representation. But this increase in time can be compromised for the 4.36%, 3.09% and 2.52% higher accuracy provided by HGSIR over Rect, Tri and Cir approaches respectively, for the 15-scene image dataset.

Another point of interest is the comparison between training and test time. The number of training images for 15-scene image dataset is 1500 and there are 2985 test images, for first two values of visual vocabulary size we observe that testing time is more as compared to training time. It should be note that the training phase besides histogram construction involves the visual vocabulary construction and cross-validation that consumes significant fraction of time. The increase in the size of visual vocabulary significantly increases the training time thereby limiting the impact of training and test dataset image ratio.

Table 10 shows the training and test time for UCM image dataset for visual vocabulary of different sizes. It confirms to our observation that the training and test time increase with increase in the size of visual vocabulary. Here we can see that the training time for HGSIR is more compared to the complementary approaches, but this time can be easily compromised for the outstanding performance of HGSIR. The training and test ratio for UCM image dataset is 0.8:0.2. Hence the training time is more compared to test time for all values of vocabulary size.

**Table 10:** Time comparison for UCM image dataset. *K* denotes the size of visual vocabulary.

*K*	Training Time				Testing Time			
	Rect	Tri	Cir	HGSIR	Rect	Tri	Cir	HGSIR
50	362.48	390.46	393.26	948.729	51.08	50.56	50.2	155.24
100	629.28	692.1	655.415	1693.201	54.62	55.1	54.64	166.65
200	1107.497	1265.183	1582.45	4210.137	92.4	86.44	84.67	261.03
400	2888.65	2969.87	2532.525	9502.425	108.7031	101.3	98.841	301.84

Table 11 provides time comparison for the Caltech-101 image dataset. The training and test ratio for Caltech-101 iamge dataset is 0.6:0.4. Here again we see that the training cost is significantly higher. The increase in time with respect to vocabulary size is in consistence with previous experimental results.

**Table 11:** Time comparison for Caltech-101 image dataset. *K* denotes the size of visual vocabulary.

*K*	Training Time				Testing Time			
	Rect	Tri	Cir	HGSIR	Rect	Tri	Cir	HGSIR
50	3389.21	4505.369	4461.615	11638.27	1041.31	830.675	820.86	2471.15
100	7711.89	6201.36	5779.3	21103.8	1065.18	980.32	965.62	2931.19
200	9964.58	8225.24	7193.45	29892.538	1464.59	1225.36	1193.17	3670.315

Table 12 demonstrates the training and testing time for the RSSCN7 image dataset. It again confirms the observation that time is directly proportional to vocabulary size. High performance of HGSIR is a good compromise over time, compared to complementary approaches. For RSSCN image dataset the training and test image ratio is 0.5:.5, hence it can give a better comparison of training and test time. The results confirm to our observation that the training phase consumes more time compared to testing phase.

**Table 12:** Time comparison for RSSCN7 image dataset. *K* denotes the size of visual vocabulary.

*K*	Training Time				Testing Time			
	Rect	Tri	Cir	HGSIR	Rect	Tri	Cir	HGSIR
50	344.01	341.116	365.354	675.43	273.08	268.44	266.62	805.59
100	502.17	520.78	543.98	1015.494	282.622	277.235	274.615	829.43
200	854.443	911.43	885.903	1784.479	291.01	281.05	278.93	838.66
400	1960.07	2085.336	1987.959	4349.514	301.06	301.25	296.27	902.55
600	4056.7	3201.738	4783.744	7351.356	430.33	334.65	327.011	1001.02

The Table 13 shows the time for MSRC-v2 image dataset for a vocabulary of size 100. For MSRC-v2, the training to test ratio is 0.6:0.4. For each individual scheme the training time is higher compared to testing time. Though HGSIR consumes more time compared to concurrent approaches, but its outstanding and consistent performance on challenging image benchmarks demonstrate that it is highly beneficial for scene classification.

**Table 13:** Time comparison for MSRC-v2 image dataset. *K* denotes the size of visual vocabulary.

*K*	Time	Rect	Tri	Cir	HGSIR
100	Training	68.71	64.46	61.03	157.71
100	Testing	62.84	52.33	52.019	103.24

## 5 Conclusion and future direction

In this paper, we aim to propose a novel image representation that is based on hybrid geometric spatial image representation to improve the effectiveness and classification accuracy of BoF model. The image is represented in the form of visual words histograms that are computed over the geometric regions based on circular, triangular and rectangular regions. The proposed histogram representation based on HGSIR contains the semantic information computed over three different geometric regions. The final histogram constructed through the proposed research is in a higher dimensional space and this is beneficial for image representation and classification learning. SVM with hellinger kernel is used for image classification and the proposed HGSIR is evaluated on five standard image benchmarks. The proposed HGSIR approach outperforms the circular, triangular, rectangular and other state-of-the-art methods in terms of classification accuracy. In future, we aim to investigate the performance of proposed approach by using a pre-trained deep neural network with transfer learning to evaluate the geometric spatial features for the large-scale image classification and retrieval.
